# Numerical solution of the Burgers equation associated with the phenomena of longitudinal dispersion depending on time

**DOI:** 10.1016/j.heliyon.2022.e09776

**Published:** 2022-06-22

**Authors:** Calvia Yonti Madie, Fulbert Kamga Togue, Paul Woafo

**Affiliations:** a Laboratory for Environmental Modeling and Atmospheric Physics, University of Yaounde 1, P.O. Box 812, Yaounde, Cameroon; b Institute of Fisheries and Aquatic Sciences at Yabassi, University of Douala, Box 2701 Douala, Cameroon; c Laboratory of Modelling and Simulation in Engineering, Biomimetics and Prototypes, Department of Physics, Faculty of Science, P.O. Box 812, Yaounde, Cameroon

**Keywords:** Burgers, Fourth order Runge-Kutta (RK4), Miscible fluids, Asymptotic dispersion

## Abstract

In this study, the Burgers equation governing the time-dependent dispersion phenomena is solved numerically using the finite difference scheme and the Runge-Kutta 4 algorithm with appropriate initial and boundary conditions. Two time-dependent dispersion functions have been implemented to analyze the spatio-temporal variation in the domain. For the values of K_L_ and K_A_ < 1.2 years, a significant retention of the mass of solute is observed when the dispersion function is asymptotic. The results obtained show that the concentration profiles are similar when the values of K_L_ and K_A_ ≥ 1.2 years. These results demonstrate the importance of the nature of the dispersion function on the retention capacity of solutes in the porous medium.

## 1 Introduction

The fate of properties such as salinity in underground media is of great interest due to increasing concern about the deteriorating environment and human health caused by poor quality groundwater (Ghafari et al., 2020). The transport of dissolved contaminants by heterogeneous hydrogeology is based on expressions of their functional parameters. Several processes acted simultaneously on the chemical constituents transported through the soil. This requires that quantitative descriptions of chemical transport include feasible processes such as the intensity of uptake of contaminants from the subterranean environment. Predicting the concentration of pollutants in the soil matrix is very important to minimize the risk and vulnerability of aquifers (Xie et al., 2019). In addition, the accuracy of the predicted model is crucial to adequately assess and predict the behavior of contaminant transport in the subsoil. Solute transport in groundwater systems is traditionally modeled by the classical non-linear advection-dispersion equation which can add to equilibrium uptake and first order degradation (Ding et al., 2021). The common hypothesis of the dispersion of pollutants in aquifers with constant porosity, regular infiltration flow rate and dispersion coefficient has been approached by many researchers from different angles. Here are some examples. Xue et al. (2020) relied on the new algorithm obtained by the precision of the Crank-Nicolson scheme to solve two-dimensional parabolic equations by alternating the implicit technique of direction. Kumar and Unny (1977) focussed his research on the application of Runge-Kutta methods to solve nonlinear partial differential equations using a specific fluid flow problem. Runge-Kutta of order 4 (RK4), remains up to date a very powerful tool for solving ordinary and partial differential equations. It has the advantage of being simple to program and quite stable for the current functions of physics (Kumar and Unny, 1977; Barletta et al., 2020). Most research has been directed towards improving the precision or flexibility of the classic method from Runge-Kutta methods.

Vázquez-Suñé et al. (2004) considered dispersivity as the most important and difficult parameter to assess in seawater intrusion models, in order to assess the effect of spatial variability of hydraulic conductivity on effective dispersion in seawater intrusion problems. Jaiswal et al. (2011) relied on an analytical solution of the advection-dispersion equation of an input concentration constant along an unstable horizontal flow in a semi-infinite shallow aquifer to determine the level of pollutants in groundwater. Corey et al. (1970), Warrick et al. (1971) have conducted field and experimental studies, and they have suggested that the coefficient of dispersion is not constant but increasing with time.

An unconditionally stable Crank-Nicolson finite difference scheme was used by Ravi et al. (2014) to analyze the constant and longitudinal dispersion profile of contaminants. In his studies, Ravi et al. (2014) did not take into account the time-dependent dispersions coefficients, the decay rate constant and the zero-order production rate coefficient of solute in the liquid phase as done by Das et al. (2017), Guleria et al. (2020) with a linear dispersion advection equation model. Our objective is to exploit the more stable fourth-order Runge-Kutta method (RK4) to evaluate the profile of C (X, T) contaminants through a porous medium and to conduct a comparative study between the profiles of the contaminants obtained from an asymptotic and linear dispersion coefficient, and on the other hand, from the initial and boundary conditions used in the work of Das et al. (2017). The discussion will focus on determining the dispersion parameters related to the concentration profiles using the two time-dependent dispersion coefficients.

## 2 Materials and methods

### 2.1 Physical model

**Figure 1:**
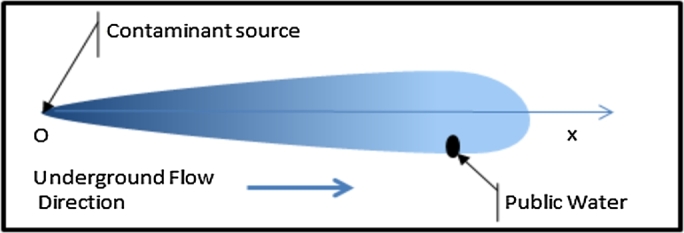
Infiltration and dispersive system of pollutants in the porous medium.

Water containing pollutants such as sewage eventually seeps into the aquifer which is a reservoir of drinking water. As water passes through the soil, pollutants are mixed, adsorbed, dispersed and diffused by the flowing stream Fig. 1. Several efforts are being made by the scientific community to develop more precise and economical models capable of predicting the transport and concentration of solutes in unsaturated soils. The transport and mixing of contaminants is governed by the advection and dispersion equation of pollutants in the terrestrial aquifer Xie et al. (2019).

### 2.2 Physical description of the time-dependent dispersion coefficient

**Figure 2:**
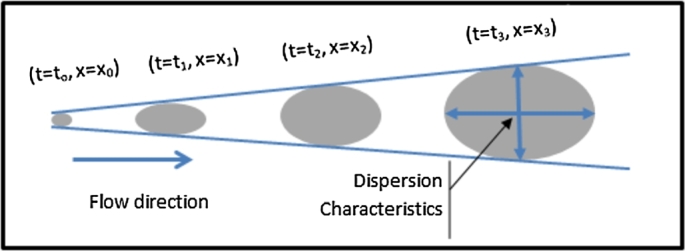
Geometry of the time-dependent dispersion problem.

Dispersion is a physical phenomenon that occurs when moving away from the injection site, the mass of solute dilutes with time to occupy an increasing volume with a correspondingly decreasing concentration see Fig. 2.

### 2.3 Mathematical model

Over the past decades, a large number of analytical and numerical solutions have been developed to estimate the fate and transport of various constituents in the underground environment. The application of these solutions is generally limited to non-variable subterranean flow fields and relatively simple initial and boundary conditions nevertheless; these solutions play an important role in contaminant transport studies, providing initial or rough estimates of the distribution of solute concentrations in soils and aquifer systems. The convection-dispersion equation has remained the basis of most analytical and numerical studies of solute transport. The dispersion equation which describes the distribution of the concentration of miscible fluids (i.e. water contaminated or salted with fresh water) in heterogeneous underground media remains one-dimensional in the direction of flow fluids along the x axis (ox) and the other oy and oz components become negligible. The transport equation can be written as equation (1) (Kajal et al., 2014; Singh et al., 2015):

(1) $$\frac{\partial C}{\partial t}+\left(\frac{1-\phi }{\phi }\right)\frac{\partial F}{\partial t}=D_{L}\frac{\partial^{2}C}{\partial x^{2}}-\nu_{0x}\frac{\partial C}{\partial x}.$$

Where C is the concentration of the contaminated fluid, F is the concentration in the solid phase, *ϕ* the porosity of the medium, with D_L_ the longitudinal dispersion coefficient on the macroscopic scale, and ($\nu_{0x}$) the component of the infiltration rate of salt water along the x-axis, and L is the length of dispersion in the direction of flow. Two cases were considered by Lapidus and Amundson (1952) for the solid phase concentration and its derivative, which are equations (2) and (3):

(2) $$F=k_{1}C-k_{2},$$

(3) $$\frac{\partial F}{\partial t}=k_{1}C-k_{2}F.$$

Here there is an equilibrium and unbalanced relationship between the concentrations in the two phases. $k_{1}$ and $k_{2}$ are the constants generally referred to as the order factor for the distribution of pollutants in aquifers. By combining the equations (3) and (2) in (1), we obtain equation (4):

(4) $$R\frac{\partial C}{\partial t}=D_{L}\frac{\partial^{2}C}{\partial x^{2}}-\nu_{0x}\frac{\partial C}{\partial x}$$

Where R is the delay factor describing the adsorption of solutes in the porous medium, its expression is equation (5):

(5) $$R=\left(1+\frac{1-\phi }{\phi }k_{1}\right).$$

The component of the infiltration rate is related to the dispersal concentration of the pollutant. Ravi et al. (2014) assumes that the rate of infiltration is $\nu_{0x}$ inversely proportional to the concentration of the pollutant as given in equation (6):

(6) $$\nu_{0x}=\frac{C(x,t)}{C_{0}}.$$

This infiltration rate is the cause of the nonlinearity in the advection – dispersion equation. $\frac{1}{C_{0}}$ represents the proportionality concentration of salt water dispersion. The new parameters of the independent variables introduced to simplify equation (4) are defined as $T=\frac{Rt}{L}$ and $X=\frac{xC_{0}}{L}$.

Therefore, equation (4) reduces to equation (7):

(7) $$\frac{\partial C}{\partial T}=D_{L}\frac{\partial^{2}C}{\partial X^{2}}-C\frac{\partial C}{\partial X}.$$

### 2.4 Description of the time-dependent dispersion model

Field and experimental evidence from studies has been suggested by Corey et al. (1970), Warrick et al. (1971), Sauty (1980), Pickens and Grisak (1981), Sposito et al. (1986) that the dispersion coefficient is not constant but apparently increasing as a function of the displacements in time or equivalently with the distance of displacement of the solute. The apparent increase in the dispersion coefficient has been called the scale effect (Fried and Combarnous, 1971). The scale effect is generally attributed to the heterogeneity of the porous formation, in particular in the heterogeneity of the hydraulic conductivity. Stochastic analyzes have shown that the dispersion depends on the transport time and increases until it reaches an asymptotic value (Gelhar et al., 1979). The theoretical deterministic analysis of Bourgeois (1984) also established that the dispersivity in a stratified aquifer is time dependent. Therefore, there is ample evidence that the dependency scale causes dispersion to vary over time. Therefore, a time-dependent dispersion model can be used to provide a rough description of the transport scale in our study by exploiting the Burger equation. In this study, a linear and asymptotic form of the time-dependent dispersion coefficient is integrated into the nonlinear PDE equation (7) to study the phenomena of longitudinal dispersion of solutes in the underground environment; this has been mentioned in various works (Guleria and Swami, 2018; Guleria et al., 2020). This type of dispersion will make it possible to better represent the results for a transport of solutes in a given porous medium. The time-dependent dispersion coefficient is studied in two forms in this work and equation (7) becomes equation (8):

(8) $$\frac{\partial C}{\partial T}=D_{L}(T)\frac{\partial^{2}C}{\partial X^{2}}-C\frac{\partial C}{\partial X}.$$

### 2.5 Time dependent linear dispersion function

The time-dependent linear dispersion increases with time without boundary conditions in the porous medium (Gao et al., 2010; Guleria et al., 2020). The linear formula for the time dependent dispersion expression in equation (8) is given by equation (9) below:

(9) $$D_{L}(T)=D_{0}\frac{T}{K_{L}}+D_{m}.$$

### 2.6 Asymptotic dispersion function depend on time

The time-dependent asymptotic distance initially increases with time and eventually approaches an asymptotic value in the porous medium (Guleria et al., 2020). The formula for time-dependent asymptotic dispersion expression in equation (8) is given by equation (10) below:

(10) $$D_{L}(T)=D_{0}\frac{T}{K_{A}+T}+D_{m}.$$

Where $D_{0}$ is the maximum dispersion coefficient for the asymptotic time-dependent and uniform dispersion function for the linear time-dependent dispersion coefficient; $D_{m}$ is the effective diffusion coefficient of time; $K_{A}$ is the time-dependent asymptotic coefficient equivalent to the mean distance travelled by the pollutants in the aquifer; $K_{L}$ is the linear coefficient depending on time. The values of the parameters of the time-dependent dispersion coefficient were determined by a sensitivity analysis of Barry and Garrison (1989). The same values of the parameters of the linear and asymptotic time-dependent dispersion coefficient exploited in the work of Guleria et al. (2020) which are also exploited in this work, are presented in the Table 1.

**Table 1:** Parameters of the time-dependent dispersion coefficient.

Parameters	Values	Parameters	Values
*D*_0_(L)	5.89 cm^2^/min	*D*_0_(A)	4.51 cm^2^/min
K_L_	4500 min	K_A_	200 min
*D*_*m*_	0 cm^2^/min	*D*_*m*_	0 cm^2^/min

**Figure 3:**
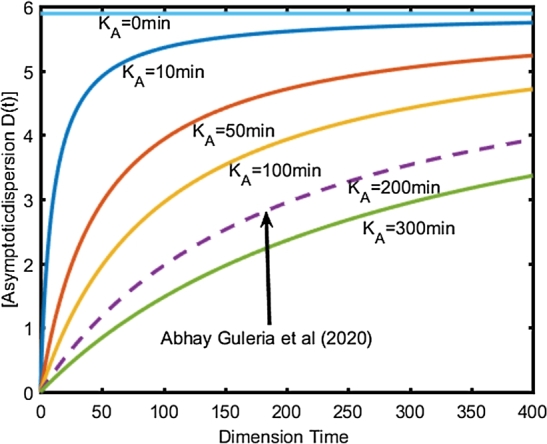
Asymptotic dispersion coefficient for various K_A_ using data from Table 1.

Fig. 3 illustrates the analysis of the asymptotic limit of the time-dependent dispersion coefficient given in relation 10, which remains important and more practical in porous media. The values of K_A_ depend on the extent of the pre-asymptotic zone. K_A_ equal to zero indicating constant dispersion. The smaller the value of K_A_, the more the dispersion approaches the asymptotic value. The values of K_A_ other than zero corresponding to the times for which the dispersion reaches half of its asymptotic value.

### 2.7 Initial and boundary conditions

**Figure 4:**
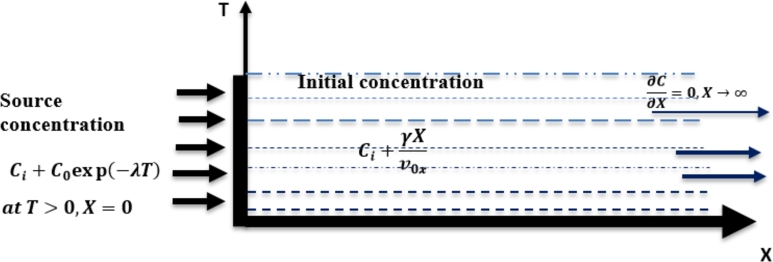
Geometry of the problem.

From the source of the pollutants, the concentration disperses more in the porous medium in the direction of flow with a front at the arrival of the boundary see Fig. 4. At the initial moment it is assumed that the aquifer is contaminated, a certain initial background concentration exists in the aquifer and it is represented by a linear combination of an initial concentration and the term of zero order production with rapid infiltration given by equation (11):

(11) $$C\left(X,0\right)=C_{i}+\frac{\gamma X}{\nu_{0x}},\;X>0,\;T=0$$

Where $C_{i}$ is the initial background concentration, $\nu_{0x}$ is flow velocity, and *γ* is the zero order production rate coefficient for liquid phase solute production. A contaminant in radioactive waste decaying exponentially with time is imposed upon entering the aquifer as a linear combination of a source concentration with an initial background concentration at the origin, to describe the transport of solutes in a natural or artificial system as expressed by equation (12) (Das et al., 2017; Singh et al., 2021):

(12) $$C(0,T)=C_{i}+C_{0}\exp(-\lambda T),\;T≻0,\;X=0,$$

*λ* is the decay rate constant and $\nu_{0x}$ is the flow velocity of fluids in the porous medium.

At the other end of the aquifer, solute transport may not be affected and therefore an exit boundary condition is prescribed as a non-flow boundary condition (Das et al., 2017). The mathematical expression of these phenomena is expressed by equation (13) as:

(13) $$\frac{\partial C}{\partial X}=0,\;X\to \infty .$$

### 2.8 Numerical solution of the mathematical model

The one-dimensional solute transport equation is a parabolic-type partial differential equation in which the finite difference technique is commonly used to obtain the numerical solution (Togue-kamga et al., 2012). An explicit finite difference technique is usually used, and ultimately results in restrictive stability criteria Das et al. (2017).

### 2.9 Fourth-order Runge-Kutta (RK4) method

The numerical technique is used to solve the equation (8) expressed as equation (14) (Togue et al., 2019; Kumar and Unny, 1977):

(14) $$\frac{\partial C}{\partial T}=f(T,C(T)).$$

The semidiscrete equation (14) is solved using the RK4 scheme according to the algorithm given by equation (15):

(15) $$\left\{ \begin{array}{cc} K_{1}=\Delta Tf(T,C(T))\; & \text{(a)}\\ K_{2}=\Delta Tf(T+\frac{1}{2}\Delta T,C(T)+\frac{1}{2}K_{1})\; & \text{(b)}\\ K_{3}=\Delta Tf(T+\frac{1}{2}\Delta T,C(T)+\frac{1}{2}K_{2})\; & \text{(c)}\\ K_{4}=\Delta Tf(T+\Delta T,C(T)+K_{3})\; & \text{(d)}\\ C(T,\Delta T)=C(T)+\frac{1}{6}(K_{1}+2K_{2}+2K_{3}+K_{4})\; & \text{(e)} \end{array}\right..$$

With (ΔT) the time step, C(T) the value of the pollutant concentration at time t and C(T + ΔT) the value of the pollutant concentration at time (T + ΔT).

The derivatives of the equation (8) are approximated by the truncated expansion of the numerical finite difference approximation scheme to determine the first and second order spatial derivatives are obtained from equations (16) and (17) (Natarajan et al., 2020):

(16) $$\frac{\partial C}{\partial X}=\frac{C_{i}^{j}-C_{i-1}^{j}}{\Delta X},$$

(17) $$\frac{\partial^{2}C}{\partial X^{2}}=\frac{C_{i}^{j+1}-2C_{i}^{j}+C_{i-1}^{j}}{\Delta X^{2}}.$$

The first order temporal discretization is given by equation (18) written as:

(18) $$\frac{\partial C}{\partial T}=\frac{C_{i}^{j+1}-C_{i}^{j}}{\Delta T}.$$

The indices (i) and (j) indicate the nodes of discretization along (X) and (T) respectively. ΔX is the spatial step. Thus, equation (8) can be written in a discrete form such as equation (19):

(19) $$\frac{\partial C_{i}^{j}}{\partial T}=f(X,T,C_{i+1}^{j},C_{i}^{j},C_{i-1}^{j}).$$

Where the function $f\left(X,T,C_{i+1}^{j},C_{i}^{j},C_{i-1}^{j}\right)$ in equation (19) is expressed as equation (20):

(20) $$f(X,T,C_{i+1}^{j},C_{i}^{j},C_{i-1}^{j})=D(j)\left(\frac{C_{i+1}^{j}-2C_{i}^{j}+C_{i-1}^{j}}{\Delta X^{2}}\right)-C_{i}^{j}\left(\frac{C_{i}^{j}-C_{i-1}^{j}}{\Delta X}\right),$$

X=iΔX, T=jΔT, with (i = 0,1,2,...,Nx) and (j = 0,1,2, ...,Nt).

The discretization of boundary and initial conditions is necessary to apply this method. The discrete equation of the initial condition and boundary conditions associated with equation (11), (12) and (13) is expressed as equation (21):

(21) $$\left\{ \begin{array}{ccc} C_{i}^{0}=C_{i}+\frac{\gamma X_{i}}{v_{0x}},\; & i≻0,\;j=0,\; & \text{(a)}\\ C_{i}^{0}=C_{i}+C_{0}\exp(-\lambda T_{j}),\; & j≻0,\;i=0\; & \text{(b)}\\ \frac{\partial C_{N_{x}}^{j}}{\partial x}=0,\; & j≻0.\; & \text{(c)} \end{array}\right.$$

### 2.10 Stability condition

Equation (8) can be written in the discrete form as equation (22) below:

(22) $$C_{i}^{j+1}=(1-2\alpha )C_{i}^{j}+\alpha C_{i+1}^{j}+\alpha C_{i-1}^{j}+\beta C_{i}^{j}(C_{i}^{j}-C_{i-1}^{j}).$$

The coefficients *α* and *β* defined in equation (22) are expressed in equation (23) and (24):

(23) $$\alpha =\frac{D_{j}\Delta T}{\Delta X^{2}}$$

(24) $$\beta =\frac{\Delta T}{\Delta X}$$

This implies that the condition of stability of equation (22) is expressed as equation (25):

(25) $$\left\{ \begin{array}{cc} 0\leq 1-2\propto \leq 1\; & \text{(a)}\\ 0\leq \propto \leq 1\; & \text{(b)}\\ 0\leq \beta \leq 1\; & \text{(c)} \end{array}\right.$$

For the conditions of stability equation (25), the coefficients *α* and *β* defined in equation (22) must be positive as expressed in equations (26) and (27), thus

(26) $$\alpha =\frac{D_{j}\Delta T}{\Delta X^{2}}≻0$$

(27) $$\beta =\frac{\Delta T}{\Delta X}≻0$$

From equation (25), we obtain the stability condition which is expressed as equation (28):

(28) $$0\leq \Delta T\leq \frac{\Delta X^{2}}{2D_{j}}.$$

These inequalities fix a strict maximum limit to the size of the time step and represent a serious limitation for the centered finite difference diagram.

## 3 Results and discussion

### 3.1 The spatial and temporal variation in the concentration

**Figure 5:**
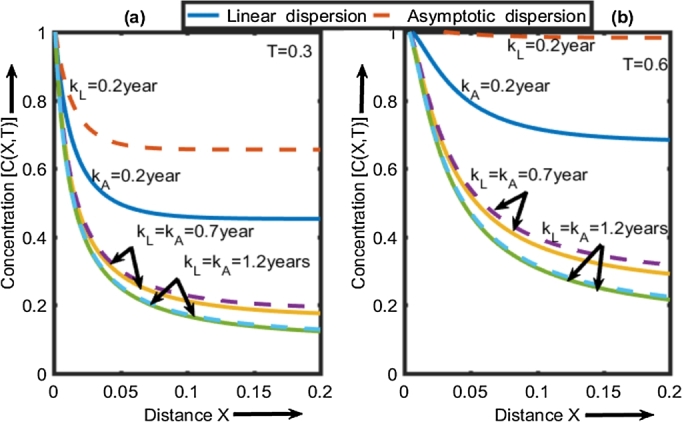
Spatial representation of the concentration C (X, T) for de T = 0.3 (a) and T = 0.6 (b) according to the different values of K_L_ and K_A_.

We analyzed the numerical solutions using the following model parameters: $C_{i}$ = 0.01, $D_{0}=0.1{\text{ km}}^{2}/\text{year}$ *λ* = 0.001 $v_{0x}$ = 0.01 km/year, γ=0.0007 (Das et al., 2017; Guleria et al., 2020; Natarajan, 2016). Figs. 5a and 5b illustrate the spatial and temporal variation in the concentration of the contaminant for different values of $K_{A}$ and $K_{L}$ (0.2,0.7,1.2,1.7years).

Fig. 5a illustrates the spatial distribution of the concentration of pollutants by considering the different values of $K_{L}$ and $K_{A}$. The spatial concentration profiles decrease exponentially in the aquifer for the values of $K_{L}$ and $K_{A}$ (0.2 years, 0.7 years, and 1.2 years). This profile decreases rapidly when the dispersion function is asymptotic, reflecting greater retention of the mass of solute in the medium. These results are similar to those obtained by Guleria et al. (2020). These authors have studied time moments (concentration per unit time) to interpret the behavior of the solute plume in porous media such as porous media laminated hydraulically with a dispersion as a function of time. The different concentrations all decrease together from the entry of the aquifer independently of the dispersion coefficient, to reach horizontal asymptotes at the different positions X = 0.029, 0.054, 0.058 respectively for three values of $K_{A}$ (0.2 years, 0.7 years, 1.2 years). The percentage of residual concentration associated with these positions is 0.76; 0.30; 0.24. On the other hand, the different horizontal asymptotes are rather obtained at the different positions X = 0.04, 0.055, 0.058 respectively for the values of $K_{L}$ ($K_{L}$ = 0.2 years, 0.7 years, 1.2 years). The associated residual concentration percentage is 0.55; 0.28; 0.24. These positions grow with the increase of $K_{L}$ and $K_{A}$. The percentage of residual solute concentration remains low when the dispersion function is asymptotic. These results confirm that the increase in $K_{A}$ assigned to asymptotic time-dependent dispersion coefficients significantly reduces the mass of solute in the subterranean medium compared to $K_{L}$ associate with linear dispersion coefficients. The concentration profiles in the porous medium get closer when the characteristic $K_{L}$ and $K_{A}$ of the medium increase. For the values of $K_{L}$ and $K_{A}<1.2$ years, a significant retention of the mass of solute is observed when the dispersion function is asymptotic. A similarity of the concentration profiles is observed for the value of $K_{L}$ and $K_{A}\geq 1.2$ years. This similarity of the concentration profile is due to the temporal variation of the effective dispersion coefficient without upper limit in the underground medium (Guleria et al., 2020).

Fig. 5b shows that the retention of the mass of solute gradually decreases compared to that observed in Fig. 5a. Likewise, for the values of $K_{L}$ and $K_{A}<1.2$ years, a significant retention of the mass of solute is observed when the dispersion function is asymptotic. Also, an analogy of the concentration profiles is observed for the value of $K_{L}$ and $K_{A}\geq$1.2 years.

## 4 Conclusion

In this study, the Burgers equation associated with a linear and asymptotic time-dependent dispersion function was numerically simulated to determine the spatiotemporal variation in concentrations. Analysis of the results shows that the concentration profiles decrease rapidly when the dispersion function is asymptotic, reflecting greater retention of the mass of solute in the medium. Values of K_L_ and K_A_ for which the concentration profiles are similar were determined. These results demonstrate the importance of the nature of the dispersion function on the retention capacity of solutes in the porous medium.

## Declarations

### Author contribution statement

Calvia Yonti Madie: Performed the experiments; Contributed reagents, materials, analysis tools or data; Wrote the paper.

Fulbert Kamga Togue, Paul Woafo: Conceived and designed the experiments; Analyzed and interpreted the data.

### Funding statement

This research did not receive any specific grant from funding agencies in the public, commercial, or not-for-profit sectors.

### Data availability statement

Data will be made available on request.

### Declaration of interests statement

The authors declare no conflict of interest.

### Additional information

No additional information is available for this paper.
